# A Cross-Sectional Study on the Role of a Lab Test Screening Program in Defining Cardiovascular Disease Risk Prevalence

**DOI:** 10.3390/jpm14030284

**Published:** 2024-03-04

**Authors:** Antoanela Curici, Mihaela Roxana Popescu, Viviana Adriana Pîrvuleț, Gabriela-Irina Marinescu, Ana Corina Ionescu

**Affiliations:** 1 Department of Cellular and Molecular Biology and Histology, “Carol Davila” University of Medicine and Pharmacy, 050474 Bucharest, Romania; viviana.pirvulet@umfcd.ro; 2 Synevo Romania, 021408 Bucharest, Romania; 3 Department of Cardiology, “Carol Davila” University of Medicine and Pharmacy, 011461 Bucharest, Romania; roxana.popescu@umfcd.ro; 4 Elias University Emergency Hospital, 011461 Bucharest, Romania; 5 Nuffield Department of Population Health, University of Oxford, Oxford OX3 7LF, UK; 6 Department of Oncological Radiotherapy and Medical Imaging, “Carol Davila” University of Medicine and Pharmacy, 020021 Bucharest, Romania; 7 Department of Radiotherapy, Colțea Clinical Hospital, 030167 Bucharest, Romania

**Keywords:** CVD risk factors, laboratory tests, screening, BMI, blood pressure

## Abstract

Recent epidemiologic studies carried out in Romania confirmed an ascending trend for cardiovascular disease (CVD) risk factor prevalence such as diabetes mellitus (DM), obesity and dyslipidemia. The aim of this study is to describe the CVD risk factor profile and preventative behavior in a representative sample of the general adult population of an Eastern Romanian urban area. More than 70% of the studied population had a body mass index (BMI) above the normal range for their age, with 36.7% of the subjects residing in obesity and severe obesity clusters. For overweight and obese subjects, the number of comorbidities (CVD, arterial hypertension and DM type 2) was higher than in the population with normal weight (44% vs. 31%, 22% vs. 14% and 18% vs. 10%, respectively). The prevalence of high blood pressure was almost double that reported in previous Romanian studies (69.3% vs. 36.6%) and higher than expected, based on self-reported known CVD diagnoses (37.5%). There was a visible difference between the results obtained for quantifiable CVD risk factors and self-reported lifestyle ones. Routine blood test monitoring may be an easy and inexpensive tool to guide educational and medical interventions to address modifiable CV risk factors in the adult population in order to prevent the fatal consequences of cardiovascular disease.

## 1. Introduction

The cardiovascular disease (CVD) prevention in clinical practice guidelines issued by the European Society of Cardiology (ESC) in 2021 state that both the incidence and mortality rate for atherosclerotic CVD have decreased in most European countries [1]. Still, it remains one of the leading causes of premature mortality and morbidity, as it is responsible for 10 million hospitalizations per year in the European Union (EU) and for more than 60 million disease-adjusted life years (DALY) losses, according to a Global Burden Disease (GBD) study [1].

According to the prevention guidelines, Romania is clustered among the very-high-risk countries, with an age-standardized incidence of CVD in 2017 of 1385 cases per 100,000 women and of 1557 cases per 100,000 men [2]. The global age-standardized death rate (ASDR) is 233.1 per 100,000 people, according to the latest published GBD data, whereas Romania has a CVD age-standardized rate of 954.8 per 100,000 inhabitants [3,4].

The European prevention guidelines support promoting a healthy lifestyle as the main strategy to prevent atherosclerotic CVD. Modifiable risk factors relevant for CVD include arterial hypertension (HTN), smoking, diabetes mellitus (DM) and plasmatic lipids containing apolipoprotein B [1].

The Strategic Planning Task Force and Statistics Committee of the American Heart Association (AHA) introduced the term “cardiovascular health” and seven health metrics. These metrics were divided into four categories: four health behaviors (smoking, diet, physical activity and BMI) and four health factors (smoking, blood pressure, total cholesterol and fasting plasma glucose). Considering the significance of quitting smoking and staying smoke-free for the promotion of health, smoking is included in the category of health behaviors as well as in that of health factors. Lifestyle factors could reduce the need for drug interventions if changes are successfully made and will reduce the relative risk of CVD as well as the increase in absolute risk associated with aging [5].

Randomized clinical trials, as well as longitudinal and epigenetic studies, demonstrate that high blood pressure (HBP) is responsible for 9.4 million deaths globally and 7% of DALYs [1]. The risk of death due to coronary artery disease (CAD) or stroke increases linearly with systolic blood pressure values of 90 mmHg or greater and diastolic blood pressure values of 75 mmHg or greater [1]. Almost half of premature deaths among smokers are due to atherosclerotic CVD [1].

Data from the National Institute for Public Health (NIPH) acknowledge that the risk factors accounting for more than 10% of CVD-related deaths in Romania are HTN (31.8%), smoking (16.3%), high cholesterol (14.4%), obesity (13.9%) and alcohol consumption (12.4%) [4]. Also, DM (type 1, type 2 and pre-diabetes states) diagnosis represents an independent risk factor for atherosclerotic CVD [1].

Recent epidemiologic studies carried out in Romania confirmed an ascending trend for DM, obesity and dyslipidemia prevalence as CVD risk factors [6,7]. Between 2007 and 2012, HTN awareness increased by 57% among the Romanian hypertensive population [8].

As there is still a wide gap between the diagnosis, treatment and control rates of CVD risk factors among the Romanian population, the need for awareness campaigns and increasing access to diagnosis remains a public health challenge.

The aim of this study is to describe the CVD risk factor profile and preventative behavior in a representative sample of the general adult population of an Eastern Romanian urban area.

## 2. Materials and Methods

### 2.1. Study Population

Between September and December 2022, a random sample of the adult population at risk for CVD was recruited out of the residents of the largest urban area in Eastern Romania. The study sample was not representative at a national level, yet, despite the small sample size, our findings may be applicable to a broader population. There are known differences in eating behaviors between different regions in Romania, as well as differences between rural and urban areas regarding access to health care providers and medical information. However, these populations share similar risk factors and demographics.

This cross-sectional study was developed through the collaboration of the regional network of family doctor practices and a privately owned chain of medical laboratories and diagnostic services with national representativeness. Data collection and blood sample collection were performed at a Synevo blood testing point in Iasi through a single study visit, in accordance with the cross-sectional design of the study, and the sample size was 2000 subjects. The Synevo blood testing point was selected due to the location’s accessibility for the population, the adequate testing space for a large number of people and the personnel capacity.

All participants provided written consent before any study procedure being performed and the patients participated anonymously in this study.

### 2.2. Data Collection

During the single-study examination, eligible subjects who were willing to participate in the study received a questionnaire concerning socio-demographic data, medical and familial history, other cardiovascular risk factors and BP-preventive behavior, and they underwent anthropometric measurements (weight and height) and BP measurements before blood sample collection. The information concerning the methodology for the variables measured and the blood tests performed is detailed in Table 1 below.

The cardiovascular risk factors assessed included self-reported CVD, stroke, atherosclerosis, DM, obesity and other relevant personal and familial medical history; body mass index (calculated from weight and height with the standard formula, units kg/m^2^); and blood tests, namely serum lipid profile (mg/dL), serum total cholesterol (mg/dL), serum LDL cholesterol (mg/dL), serum HDL cholesterol (mg/dL), serum triglycerides (mg/dL) and plasma glucose (mg/dL). The laboratory workup used for blood test processing is described below in Table 2.

The variables collected for evaluating preventive behaviors included lifestyle factors, such as diet, smoking, sedentarism, alcohol consumption and BP measurement habits.

Although most health metrics were measured reliably and objectively, the lifestyle factor data in the study population may be subject to reporting bias because they were based on self-reporting.

### 2.3. Statistical Analysis

A statistical analysis was performed using IBM SPSS Statistics 29.0 software at a significance level of *p* < 0.05.

A descriptive analysis was performed for continuous data (means, medians, standard deviations) and for categorical data (frequency analysis).

A normality test of the continuous data distribution was performed using the Kolmogorov–Smirnov test and the Shapiro–Wilk test in order to allow for the selection of the appropriate tests for performing an inferential statistical analysis. Pearson’s correlation coefficient was used to assess the relationships between continuous data. The independent *t*-test was used for the analysis of the differences between continuous and categorical data, and a one-way ANOVA allowed for an analysis of the mean differences between variables.

The selection of the statistical methods was mainly driven by the research goal, which is to identify differences and describe trends and potential relationships among the study variables. For the study sample size and statistical power, both a *t*-test and an ANOVA are preferred.

## 3. Results

Out of the 2000 people invited to participate in the study, there were 1396 subjects who provided their written informed consent. Data from 1289 subjects were complete and eligible, according to the study design, and entered the analysis.

**Figure 1:**
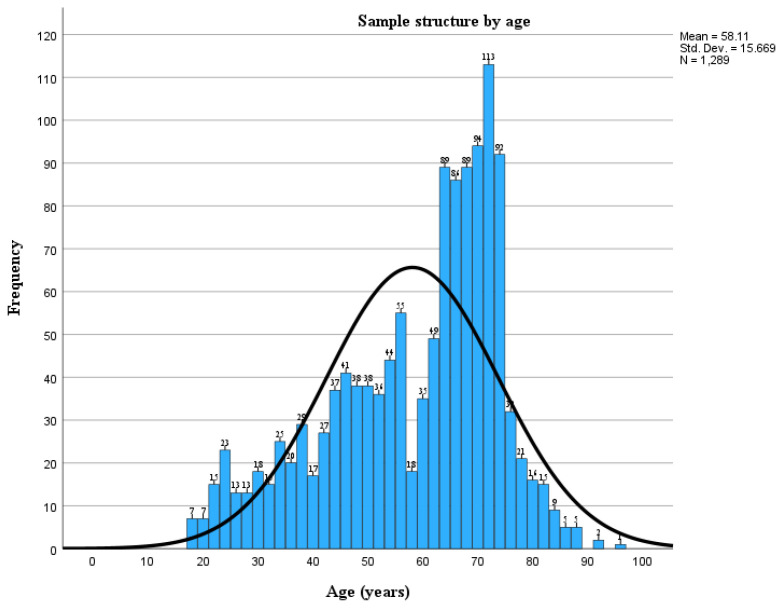
Sample structure by age.

The sample has a non-normal distribution for age (Figure 1), as confirmed by testing for normality, with a difference between the mean (58.11 years) and median (63.00 years), skewness to the left (skewness coefficient −0.686), a lower peak around the mean and a Kolmogorov–Smirnov test and Shapiro–Wilk test significance of <0.001.

The sample analysis by gender showed a 2.2:1 distribution favoring female subjects with no significant differences between the mean age of the groups: 57.81 (±15.65) for women and 58.78 (±15.69) for men.

More than half (64.1%) of the study population were over 55 years of age, and 73.62% of the subjects weighted above the range for a normal weight, which contrasted with the self-reported medical history of obesity of 16.9% (Table 3).

The definitions for diabetes mellitus, high blood pressure, obesity and dyslipidemia thresholds for LDL cholesterol respected the most current version of the diagnosis and monitoring guidelines [9,10,11,12].

The average values of the measured variables in the blood tests (plasma glucose, total cholesterol, serum LDL cholesterol, serum TG) were above the guideline threshold for maintaining a low CV risk, and 41% of the subjects had at least one lifestyle risk factor present.

The cut-offs for BMI and blood pressure, as well as DM and HTN diagnosis, were evaluated by age group, similar to the age clusters used in other epidemiological studies concerning CVD risk factors in Romania [8,9,10,11,12,13].

Strong positive correlations were identified between LDL-C and serum cholesterol; total lipids and serum cholesterol; and LDL-C and total lipids using Pearson’s correlation coefficient. A moderate positive correlation was identified between the SBP and DPB values, and low positive correlations were obtained between the BMI and triglycerides values; serum cholesterol and triglycerides; SBP and age; BMI and age; and SBP and BMI.

An inverse relationship between the levels of triglycerides and HDL-C, as well as between BMI and HDL-C, as highlighted by the negative correlation coefficients, was identified (Table 4). All other CVD risk factors were either very slightly or negligibly correlated between themselves.

**Figure 2:**
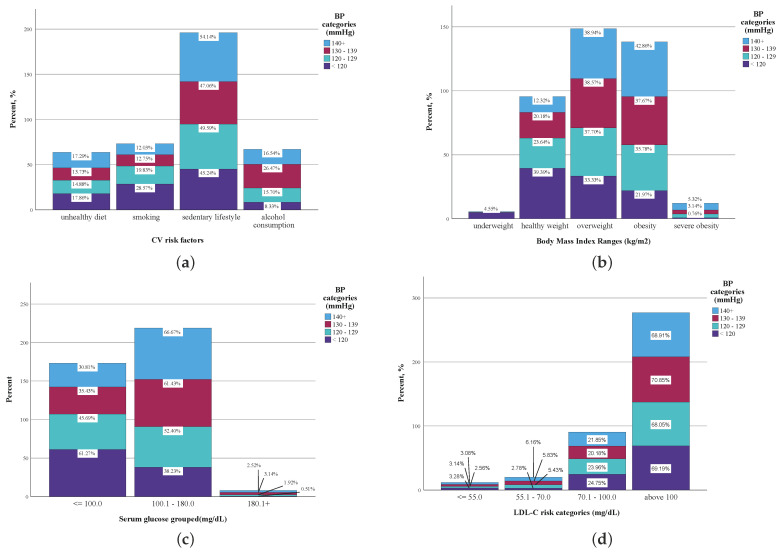
CV risk factor frequencies by ESC/ESH SBP categories. (**a**) Self-reported lifestyle CV risk factor distribution by SBP risk categories (n = 524). (**b**) BMI distribution by SBP risk categories (n = 1289). (**c**) Plasma glucose levels by SBP risk categories (n = 1289). (**d**) Serum LDL-C levels by SBP risk categories (n = 1289).

As a positive correlation between SBP and DBP has been demonstrated, a CV risk factor prevalence analysis was performed as a function of the SBP sample distribution, in accordance with the most recent version of the International Society of Hypertension (ISH) guidelines (Figure 2). The frequencies (percentage) of the self-reported lifestyle CV risk factors (Figure 2a), the BMI group distribution (Figure 2b), the plasma glucose levels (Figure 2c) and the serum LDL-C levels were calculated for each of the four SBP categories (Figure 2d): normal SBP, < 120 mmHg; elevated SBP, between 120 and 129 mmHg; stage I hypertension, SBP between 130 and 139 mmHg; and stage II hypertension, SBP ≥ 140 mmHg [14].

More than half of the subjects reporting a sedentary lifestyle (54.14%) had SBP values ≥ 140 mmHg, and 87.12% of the subjects with SBP values corresponding to stage II hypertension (SBP ≥ 140 mmHg) had a BMI above the normal BMI values. Also, two out of three subjects with grade II hypertension recorded plasma glucose levels > 100 mg/dL, and approximately two-thirds of the subjects had serum LDL-C values > 100 mg/dL irrespective of the SBP classification of normal, elevated or hypertension grades.

**Figure 3:**
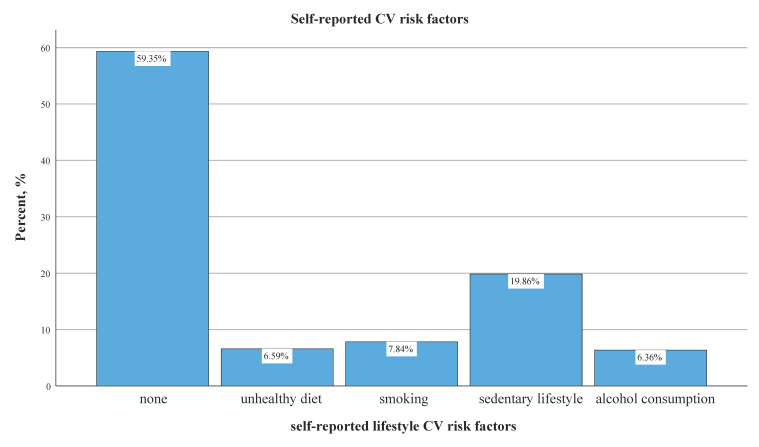
Self-reported lifestyle CV risk factors and preventive behavior by BP categories.

At least one self-reported lifestyle CV risk factor (unhealthy diet, smoking, sedentary lifestyle or alcohol consumption) was recorded in 40.7% of the subjects (Figure 3). The only lifestyle CV risk factor with a double-digit representation was sedentarism, with a reported frequency of 19.86%. Smoking, unhealthy diet and alcohol consumption were each reported in less than 10% of cases by the study population.

**Figure 4:**
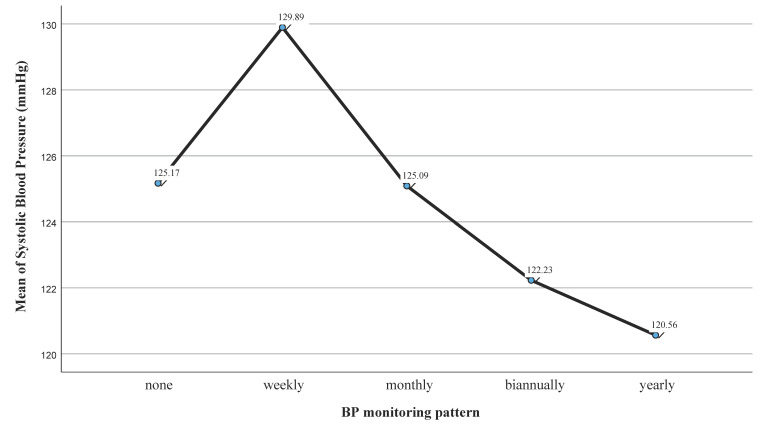
Mean plot of mean SBP (mmHg) based on BP monitoring patterns.

A one-way analysis of variance was conducted to evaluate the null hypothesis that there is no difference in the systolic blood pressure values (mmHg) of the subjects based on their BP monitoring patterns (N = 1289). The independent variable, BP monitoring patterns, included five groups: weekly (M = 129.89; SD = 19.4; n = 539), monthly (M = 125.09; SD = 18.17; n = 283), biannually (M = 122.23; SD = 18.50; n = 174), yearly (M = 120. 56; SD = 18.85; n = 216) and never (M = 125.17; SD = 18.81; n = 77). The assumption of normality was evaluated using histograms and found to be tenable for all groups. The assumption of the homogeneity of variances was tested and found to be tenable using Levene’s test: F (4,1284) = 0.302 and *p* = 0.877. There was a significant difference among the patterns of BP monitoring according to the SBP measurements (F (4,1284) = 12.10; *p* < 0.001; partial eta squared: 0.036), thus demonstrating the significant differences in SBP (mmHg) based on BP monitoring patterns (Figure 4). Post hoc comparisons (Tukey’s HSD test, tenable equal variances) were conducted to evaluate pairwise differences among the group means. The tests exposed significant pairwise differences between the mean scores of SBP monitored weekly and the mean scores of SBP monitored monthly (*p* = 0.005), biannually (*p* < 0.001) and yearly (*p* < 0.001).

To describe the CV health preventive behavior within the study sample, an independent-sample *t*-test was conducted to compare the values registered for measurable CV risk factors (blood tests) for subjects with a normal SBP (<120 mmHg) against those of subjects with an elevated SBP (≥120 mmHg) and for subjects with a normal BMI (<25 kg/m^2^) against those with increased BMI values (≥25 kg/m^2^).

Table 5 and Table 6 summarize the independent *t*-test results for the two comparisons, reporting the mean, standard deviation, statistical significance, degrees of freedom, mean difference and standard error differences for each of the blood test results.

The magnitude of the difference in the means between the normal and elevated SBP groups was significant for HDL-C, serum cholesterol, plasma glucose, triglycerides and total lipid levels, but it did not reach significance for LDL-C (Table 5).

The magnitude of the difference in the means between the normal and elevated BMI groups was significant for LDL-C, HDL-C, plasma glucose, triglycerides and VLDL-C levels, but it did not reach significance for serum cholesterol or total lipid levels (Table 6).

## 4. Discussion

This study provided new insights concerning CV risk factors’ prevalence in an urban population from a Romanian region, as well as regarding preventive behavior within the studied sample.

The CVD burden is high for Romania; 35% of yearly recorded deaths are due to CVD, and Romania has the highest rate of stroke mortality in Europe [4,15]. Increasing the population’s awareness about their health status and modifiable health factors is among the critical strategies for CVD primary and secondary prevention. Health promotion and cardiovascular prevention should acquire central roles in health policies. Public health campaigns play a vital role in raising awareness about cardiovascular risk factors and promoting preventive behaviors.

The mean BMI in the study was consistent with other epidemiological studies previously carried out in Romania and confirmed that, on average, the Romanian adult population has BMIs outside the BMI range corresponding to normal health [16,17,18,19]. More than 70% of the studied population had a BMI above the normal range for their age, with 36.7% of the subjects residing in the obesity and severe obesity clusters. For the overweight and obese subjects, the number of comorbidities related to CVD risk (CVD, arterial hypertension and DM type 2) was higher than in the population with a normal weight (44% vs. 31%, 22% vs. 14% and 18% vs. 10%, respectively). For the quantifiable CVD risk factors, all but total cholesterol and total lipids were significantly higher in subjects with a BMI above the normal range, compared with the study sample with normal BMIs.

Diet and exercise control, together with specific medical therapies, are necessary following the onset of obesity or lifestyle disorders. Even so, a lot of patients are unaware of this. Following cardiovascular disorders, the best possible pharmacological and/or interventional therapy must be combined with a proper diet and exercise. Some patients, nevertheless, struggle to regulate how much they eat and how active they are.

The prevalence of high blood pressure was almost double that reported in previous studies with national representativeness for a Romanian territory (69.3% vs. 36.6%) and higher than expected based on the self-reported known CVD diagnoses (37.5%) [19]. Although one of the most obvious reasons for this is the lower cut-off values for defining HTN in the current clinical practice guidelines, it can also be assumed that there is a low level of awareness among the population regarding their blood pressure range. While less than 1% of the studied population reported never measuring their BP, 30% of the subjects are checking their BP values either twice per year or annually.

There was a visible difference in the study population between the results obtained from quantifiable CVD risk factors (blood tests) and the self-reported lifestyle-related CV risk factors. The self-reported smoking rate was 7.84%, which is lower than both that reported in previous studies (e.g., 16% or 40.9%) and that from the WHO data (27%) [16,17,20].

Also, differences between the other self-reported lifestyle factors, like an unhealthy diet and alcohol consumption, seemed lower than those registered in global statistics and previous research [21]. According to a national study in Romania, over a third of adults have declared that they episodically consume alcohol in excess, at least once a month, which represents one of the highest rates in the EU (35% compared to 19% on average in the EU). There is a strong gender disparity between women and men in favor of men: 53% of the men reported such behavior, unlike women, in the case of which this behavior was reported by less than one in five women (18%). This could be an explanation for the discrepancies reported in our study [22].

The self-reported lifestyle factors were less prevalent than expected based on the mean results reported for the blood tests performed during the study, which reflect quantifiable CV risk factors.

One hypothesis for this is that the population’s awareness is still low regarding their health status and the cardiovascular risk embodied by their lifestyle, and this can be quantified through periodic assessments of CV risk through specific routine blood tests that can support timely diagnosis and treatment, although further research with solid methodology is still necessary to confirm this. Also, recall bias as well as social desirability bias may have had an influence on the self-reported data, and this cannot be ignored. The specific design of the study (single-center, limited number of investigators interacting with the study sample, collection of lab measures representative of CV risk) has the potential to mitigate the self-reporting data reliability issues without discarding them completely. The high number of subjects recruited for this study supports the relevance of the results. However, the study lacks national representativeness, which may have been obtained through a multicentric study design methodology. Further research with the aim of comprehensively assessing CV risks within the general population, both rural and urban, by examining various factors such as lifestyle, medical history and socioeconomic status may be needed in order to provide valuable insights into the prevalence, distribution and causes of CV risks. This type of comprehensive research should be supported by public healthcare interest and could have the scope to inform public health strategies and interventions aimed at reducing CVD burden on a national level.

Another limitation of the study is the high number of self-reported variables without proper medical documentation to support the accurate CV risk profile of the study subjects at the moment of enrollment. In particular, proper documentation of their medical history containing details of their current medication and hospitalization history would have allowed a comprehensive evaluation of the disease burden; however, this was outside the scope of the current study.

There are other confounding factors that were not considered and that could be factored in as modifiable lifestyle CV risk factors, like mental health status, socioeconomic status, level of education and access to information and routine medical care. Collecting a broad amount of data was outside the scope of this study. Obtaining a comprehensive description of the CVD factor profile requires the inclusion of confounding factors, and further research is thus encouraged to complete this endeavor.

## 5. Conclusions

According to this research, most individuals within the study population had poor cardiovascular health. Consistent effort, particularly in supporting lifestyle changes, will be necessary on individual, population and social levels to improve CV health. This should start at a younger age, through parental and societal education about a healthy lifestyle.

Routine blood test monitoring performed within the scope of identifying objectifiable CV risk factors may be an easy and inexpensive tool to guide and inform individualized educational and medical interventions that address modifiable CV risk factors in the adult population in order to prevent the fatal consequences of cardiovascular disease. Also, this type of program may be a means of raising awareness about other unperceived CV risk factors, like being overweight, that might also impact the future burden of disease for these patients.

Accessibility to healthcare services is crucial for the early detection and management of cardiovascular risk factors. This includes access to primary care providers, specialists, diagnostic tests, medications and lifestyle intervention programs. Efforts to improve healthcare access, particularly in underserved communities, can help reduce health disparities and improve cardiovascular outcomes.

Tracking CV risk throughout life is important, both on an individual and societal level, since both CV risk and preventive measures are dynamic and continue with aging and the accumulation of comorbidities. Epidemiologic data suggest that ideal CV health is linked to a decreased risk of CVD, decreased rates of both CVD and all-cause mortality, improved quality of life, disease-free survival and decreased healthcare expenses [5].

## Figures and Tables

**Table 1:** Equipment and units for measured study variables.

Measured Variable	Measurement Device	Unit (±Error)
**weight**	electronic scale, calibrated	kg (±0.1)
**height**	stadiometer	cm (±0.5)
**blood pressure**	certified oscillometric-based automatic office BP measurement, calibrated, cuff width adjusted to arm circumference	mmHg (±3)

**Table 2:** Laboratory specification for blood tests.

Blood Test	Methodology/Reagent/Manufacturer/Analyzer	Methods’ Analytical Characteristics					
		BIAS		Within-Run Imprecision		Between-Run Imprecision	
		N	P	N	P	N	P
**Plasma glucose**	UV enzymatic/hexokinase/GLUC3 Roche/Cobas 8000 c702 module (Roche Diagnostics GmbH, Mannheim, Germany)	−0.84	−3.12	0.7227	0.5487	2.2851	1.8003
**Serum total cholesterol**	Enzymatic/colorimetry/CHOL2/Roche/Cobas 8000 c702 module	3.1956	5.636	0.6555	0.7504	3.1551	2.2071
**Serum LDL-cholesterol**	Enzymatic colorimetry/LDLC3/Roche/Cobas 8000 c702 module	−1.788	−0.392	0.4596	0.8032	1.6401	2.6374
**Serum HDL-cholesterol**	Enzymatic colorimetry/HDLC4/Roche/Cobas 8000 c702 module	−0.768	−0.592	0.277	0.407	1.993	1.099
**Serum triglycerides**	Enzymatic/colorimetry/TRIGLR/Roche/Cobas 8000 c702 module	1.736	2.604	0.319	0.438	2.175	1.459

**Table 3:** Descriptive characteristics of study population.

	Total	Male	Female
	N = 1289	N = 400 (31.03%)	N = 889 (68.97%)
**Age groups (years), n (%)**			
18–35	136 (10.6)	36 (9)	100 (11)
35–54	327 (25.4)	110 (28)	217 (24)
55–74	720 (55.9)	211 (53)	509 (57)
75+	106 (8.2)	43 (11)	63 (7)
**Anthropometric measurements, mean (±SD)**			
BMI, kg/m^2^	28.69 (±5.64)	28.85 (±4.83)	28.62 (±5.97)
Systolic blood pressure, mmHg	125.96 (±19.23)	128.52 (±18.07)	124.81 (±19.63)
Diastolic blood pressure, mmHg	72.68 (±10.87)	73.85 (±10.66)	72.15 (±10.93)
**Blood tests, mean (±SD)**			
Total cholesterol, mg/dL	194.21 (±45.25)	188.73 (±47.98)	196.68 (±43.76)
LDL-C, mg/dL	123.54 (±41.38)	121.86 (±42.90)	124.29 (±40.68)
HDL-C, mg/dL	54.31 (±15.02)	47.84 (±14.72)	57.22 (±14.24)
VLDL-C *, mg/dL	27.24 (±15.32	30.69 (±19.16)	25.69 (±12.95)
Total lipids, mg/dL	662.84 (±142.76)	668.04 (±164.81)	660.51 (±131.67)
Triglycerides, mg/dL	136.15 (±76.57)	153.35 (±95.81)	128.42 (±64.71)
Serum glucose, mg/dL	108.79 (±27.27)	112.26 (±30.83)	107.22 (±25.37)
**Comorbidities, n (%)**			
CVD	483 (37.5)	150 (37.5)	333 (37.5)
Stroke	55 (4.3)	15 (3.8)	40 (4.5)
DM1	39 (3.0)	12 (3.0)	27 (3.0)
DM2	183 (14.2)	64 (16)	119 (13.4)
Obesity	150 (16.9)	67 (16.8)	217 (16.8)
Dyslipidemia	220 (17.1)	50 (12.5)	170 (19.1)
Atherosclerosis	30 (2.3)	9 (2.3)	21 (2.4)
Other **	101 (7.8)	20 (5.0)	81 (9.1)
**Risk factors, n (%)**			
Unhealthy diet	128 (.9)	41 (10.3)	87 (9.8)
Smoking	161 (12.5)	54 (13.5)	107 (12)
Sedentary lifestyle	274 (21.3)	87 (21.8)	187 (21.0)
Alcohol	84 (6.4)	67 (16.8)	17 (1.9)
**Familial medical history, n (%)**			
CVD	492 (38.2)	130 (32.5)	362 (40.7)
Stroke	172 (13.3)	38 (9.5)	134 (15.1)
DM	259 (20.1)	73 (18.3)	186 (20.9)
Obesity	129 (10.0)	24 (6.0)	105 (11.8)
Atherosclerosis	38 (2.9)	5 (1.3)	33 (3.7)

* calculated. ** other relevant comorbidities included thyroid, renal and endocrine pathology, cancer and autoimmune disorders.

**Table 4:** Correlation coefficients (Pearson’s r) of the cardiovascular risk factors.

	Age	HDL-C	LDL-C	Serum cholesterol	TG	VLDL-C, calculated	Total lipids	SBP	DBP	BMI
**Age**		−0.048	−0.041	−0.015	0.136 **	0.136^**^	0.061 *	0.376 **	0.100 **	0.271 **
**HDL-C**	−0.048		0.021	0.219^**^	−0.440 **	−0.440 **	−0.079 **	−0.088 **	−0.072 **	−0.285 **
**LDL-C**	−0.041	0.021		0.950 **	0.175 **	0.175 **	0.762 **	0.035	0.096 **	−0.005
**Serum cholesterol**	−0.015	0.219 **	0.950 **		0.259 **	0.259 **	0.854 **	0.055 *	0.106 **	−0.041
**TG**	0.136 **	−0.440 **	0.175 **	0.259 **		1.000 **	0.720 **	0.179 **	0.183 **	0.225 **
**VLDL-C**	0.136 **	−0.440 **	0.175 **	0.259 **	1.000 **		0.720 **	0.179 **	0.183 **	0.225 **
**Total lipids**	0.061 *	−0.079 **	0.762 **	0.854 **	0.720 **	0.720 **		0.134 **	0.172 **	0.091 **
**SBP**	0.376 **	−0.088 **	0.035	0.055 *	0.179 **	0.179 **	0.134 **		0.517 **	0.278 **
**DBP**	0.100 **	−0.072 **	0.096 **	0.106 **	0.183 **	0.183 **	0.172 **	0.517 **		0.179 **
**BMI**	0.271 **	−0.285 **	−0.005	−0.041	0.225 **	0.225 **	0.091 **	0.278 **	0.179 **	

*. Correlation is significant at the 0.05 level (2-tailed) **. Correlation is significant at the 0.01 level (2-tailed).

**Table 5:** Differences in blood test results between subjects with normal and elevated SBP (mmHg, N = 1289).

Blood Tests (mg/dL)	SBP (mmHg)	N	Mean	SD	Std. Error Mean	Levene’s Test for Equality of Variances		*t*-Test for Equality of Means						
						F	Sig.	t	df	Sig. (2-tailed)	Mean Diff.	Std. Error Diff.	95% CI of the Difference	
													Lower	Upper
LDL-C	≥ 120	893	124.57	42.78	1.431	7.12	0.008	1.39	845.59	0.164	3.325	2.386	−1.359	8.009
	< 120	396	121.24	37.99	1.909									
HDL-C	≥ 120	893	53.46	15.03	0.503	1.27	0.260	−3.08	1287.00	0.002	−2.786	0.904	−4.560	−1.012
	< 120	396	56.24	14.87	0.747									
Serum cholesterol	≥ 120	892	195.88	46.34	1.552	3.59	0.058	1.97	1286.00	0.048	5.390	2.729	0.036	10.745
	< 120	396	190.49	42.51	2.136									
Plasma glucose	≥ 120	893	112.27	27.86	0.932	14.60	0.000	7.40	861.81	0.000	11.345	1.532	8.338	14.352
	< 120	395	100.93	24.17	1.216									
TG	≥ 120	893	145.03	80.04	2.679	6.45	0.011	6.91	936.41	0.000	28.877	4.179	20.676	37.078
	< 120	396	116.15	63.83	3.207									
Total lipids	≥ 120	893	675.29	145.89	4.882	2.46	0.117	4.94	834.66	0.000	40.520	8.210	24.404	56.635
	< 120	396	634.77	131.36	6.601									

**Table 6:** Differences in blood test results between subjects with normal and elevated BMI (kg/m^2^, N = 1289).

Blood Tests (mg/dL)	BMI (kg/m^2^)	N	Mean	SD	Std. Error Mean	Levene’s Test for Equality of Variances		*t*-Test for Equality of Means						
						F	Sig.	t	df	Sig. (2-tailed)	Mean Diff.	Std. Error Diff.	95% CI of the Difference	
													Lower	Upper
LDL-C	≥25.1	945	124.61	42.60	1.386	10.189	0.001	1.624	682	0.105	4.00	2.461	−0.836	8.829
	<25.0	344	120.61	37.73	2.034									
HDL-C	≥25.1	945	51.70	13.57	0.442	20.489	0.000	−9.901	523	0.000	−9.81	0.990	−11.753	−7.861
	<25.0	344	61.50	16.44	0.887									
Serum cholesterol	≥25.1	944	194.42	46.15	1.502	2.776	0.096	0.267	1286	0.789	0.76	2.851	−4.831	6.354
	<25.0	344	193.66	42.74	2.304									
VLDL (calc.)	≥25.1	945	29.65	15.82	0.514	10.741	0.001	11.185	831	0.000	9.03	0.808	7.447	10.617
	<25.0	344	20.62	11.54	0.622									
TG	≥25.1	945	148.17	79.06	2.572	10.920	0.001	11.147	831	0.000	45.00	4.037	37.072	52.918
	<25.0	344	103.17	57.70	3.111									
Total lipids	≥25.1	945	675.19	145.61	4.737	3.612	0.058	5.197	1287	0.000	46.25	8.900	28.791	63.713
	<25.0	344	628.93	128.88	6.949									
Plasma glucose	≥25.1	945	112.60	28.11	0.914	21.976	0.000	9.642	783	0.000	14.31	1.484	11.394	17.219
	<25.0	343	98.29	21.64	1.168									

## Data Availability

The data that support the findings of this study are available from the corresponding author upon reasonable request.
